# Supplementary material for: Podoconiosis: Clinical spectrum and microscopic presentations
Source: PLoS Negl Trop Dis. 2022 May 23;16(5):e0010057. doi: 10.1371/journal.pntd.0010057 (PMC9166354; doi:10.1371/journal.pntd.0010057)
Supplement: S1 Table — (DOCX) [file pntd.0010057.s001.docx]

**Annex A**

1. Clinical staging system for podoconiosis [1]

| **Stage** | **Characteristics clinical future** |
| --- | --- |
| Stage 1 | Swelling reversible overnight  The swelling is not present when the patient first gets up in the morning. Changes such as hyperpigmentation and nail dystrophy are unusual, but may be seen. The swelling is usually confined beneath the ankle. |
| Stage 2 | Below-knee swelling that is not completely reversible overnight; if present, **nodules/papules are below the ankle only**. Persistent swelling that does not reach above the knee.  Nodules/papules may take the form of dermal nodules, ridges or bands. Tourniquet-like effects may be observed at this stage or any subsequent stage, depending on the position of dermal ridges and nodules in relation to joints.  Mossy changes may be apparent, but their presence depends on a range of factors including the use of plastic footwear. Interdigital maceration and hyperpigmentation are often present at this stage, and nail dystrophy almost always present. |
| Stage 3 | Below-knee swelling that is not completely reversible overnight; **nodules/papules present above the ankle**  **Persistent swelling that does not reach above the knee.**  Dermal nodules, ridges or bands seen or felt above the ankle. Tourniquet-like effects are frequently observed at this stage  Any of the other changes mentioned for Stage 2 may also be present.  Joint mobility not affected. |
| Stage 4 | Above-knee swelling that is not completely reversible overnight; nodules/papules present at any location  **Persistent swelling that is present above the knee.**  Any of the other changes mentioned for Stage 2 may also be present. In addition, signs of lymphectasia may be apparent, particularly on the thigh. |
| Stage 5 | **Joint fixation**; swelling at any place in the foot or leg  The ankle or interphalangeal joints become fixed and difficult to flex or dorsiflex. This may be accompanied by adhesion and fusion of the toe web spaces, making the toes appear short or indistinct. Sensation is preserved. X-rays show tuft resorption and loss of bone density. |

2: Revised podoconiosis clinical staging criteria

| Stage | Characteristics clinical future |
| --- | --- |
| Stage 1 | ***Necessary:***  Foot oedema (Swelling) reversible. Pitting oedema confined to the foot appreciable in the evening and disappears after overnight rest.  ***Additional features:***  Hyperpigmented and hyperkeratotic skin change on the anterior one third of the foot (dorsum), splaying of the toe with plantar oedema. |
| Stage 2 | ***Necessary***:  Below-knee pitting swelling that is not completely reversible overnight.  ***Additional features:***  Papules, nodules **and tumors below the ankle**. Mossy changes may be apparent. Interdigital maceration, hyperpigmentation are often present at this stage, and nail dystrophy may be present. |
| Stage 3 | ***Necessary***  Below-knee persistent swelling that is not completely reversible overnight; **nodules/papules/tumors present above the ankle.**  ***Additional features:***  Fibrotic (non-pitting) swelling, toe fusion and bone resorption (toe). Oozing or lymphorrhea on the foot with maceration  Any of the other changes mentioned for Stage 1 and 2 may also be present. |
| Stage 4 | ***Necessary:***  **Above-knee persistent swelling.**  ***Additional features:***  Any of the other changes mentioned for Stage 1 to 3 may also be present. |
| Stage 5 | ***Necessary***  Fibrotic swelling at any place in the foot or leg with **joint fixation**;  The ankle or interphalangeal joints become fixed and difficult to flex or dorsiflex. This may be accompanied by adhesion and fusion of the toe web spaces, making the toes appear short or indistinct.  ***Additional features :***  Any of the other changes mentioned for Stage 2 to 4 may also be present. |

Stages category based on severity and treatment response

1. Mild stages: stage 1 and 2
2. Advanced stages: Stage 3, 4 and 5

Reference

*Tekola, F., Ayele, Z., Mariam, D. H., Fuller, C., & Davey, G. (2008). Development and testing of a de novo clinical staging system for podoconiosis (endemic non-filarial elephantiasis). Tropical medicine & international health: TM & IH, 13(10), 1277–1283. doi:10.1111/j.1365-3156.2008.02133.x*
