# Supplementary material for: Podoconiosis: Clinical spectrum and microscopic presentations
Source: PLoS Negl Trop Dis. 2022 May 23;16(5):e0010057. doi: 10.1371/journal.pntd.0010057 (PMC9166354; doi:10.1371/journal.pntd.0010057)
Supplement: S1 Text — (DOCX) [file pntd.0010057.s002.docx]

**Annex B**

Operational Definitions

Podoconiosis clinical definition

Podoconiosis is a non-communicable tropical form of lymphoedema resulting in bilateral or unilateral swelling of the lower legs (usually below-knee) and impaired skin barrier function, acquired through prolonged barefoot exposure to red clay soils of volcanic origin [1].

Types of lymphoedema [2]

(1) Soft and pitting (“waterbag” type) − subdermal oedema that is soft to the touch and pits with pressure; it has little dermal fibrosis. Usually, the swelling has a narrow neck around the knee and wider base on the foot (flask-like appearance). The skin will have a smooth and dumpy surface, with occasional lymphorrhea especially on the foot, which attracts flies. Often, there is loss of hair growth. With time, the foot and lower leg become large and flabby. With elevation there is considerable reduction in size. Swelling may lead to redundant skin folds around the ankle joint and ballooning over the toes.

(2) Hard and sclerotic/fibrotic or leathery leg ‘lymphoedema’ − sclerosis governs the changes in the skin and subcutis, which become woody hard and grossly thickened. Due to increasing hyperkeratosis, the overlying skin on the foot takes on a sandpaper-like appearance, and eventually the so-called ‘mossy’ appearance (see below).

Under areas of compression, such as a sandal strap, the skin remains smooth and dumpy. The stiff, sclerotic nature of this altered skin especially on the ankle compromises the normal flexibility of the ankle and toe which makes it vulnerable to cracking and trauma, in addition to ankylosis of the joints.

(3) Mixed lymphoedema – characterized by grossly swollen and non-pitting oedema, below the knee, not-reducible overnight and without sclerotic changes. There may be variation in the compressibility of the lymphoedema below the ankle and above the ankle.

*Hyperkeratosis/ mossy foot:* A sandy appearance or feel of the skin with rough (warty) coalescing papules in a slipper distribution, predominantly on the base of the toes (dorsum), and lateral part of the foot [1]

Acute Dermato-Lymph-Angio-adenitis (ADLA):

An acute presentation of fever, chills, rigor, headache, and pain in the affected leg. On examination the affected extremity is diffusely swollen, red/violaceous, tense, warm, and can be tender. Red streaks may be visible along the inflamed lymphatics. Edematous inflammatory plaques, clearly demarcated from normal skin, are seen. Late-stage ADLA is characterised by superficial exfoliation of the skin in the affected area. Inguinal lymph nodes may be tender and enlarged [3]. ADLA can be considered a variant of erysipelas complicated by the chronic conditions of podoconiosis.

### Signs of fungal infection*:*

### Erythema, maceration, fissuring, whitish*,*curd*-*like patches and scaling, most often in the interdigital area, but also between redundant skin folds and nodules. Some extension of the whitish scaling onto the plantar surface of the foot may occur. Some may have itching.

*Reference*

*Price E (1990) Podoconiosis:Non-filarial Elephantiasis. Oxford: Oxford Medical.*

1. *Tekola-Ayele F, Embiale WY. Podoconiosis: tropical lymphedema of the lower legs. In: Dermatology and Allergology—Principles and Practice. 1st ed. Hong Kong: iConcept Press Ltd; 2014; 201. https://pdfs.semanticscholar.org/df19/c491c922dba13a894cc8f87db4baec5df89d.pdf.*
2. *Goel T.C., Goel A. (2016) Acute Dermatolymphangioadenitis (ADLA). In: Lymphatic Filariasis. Springer, Singapore. https://doi.org/10.1007/978-981-10-2257-9_15*
