# Supplementary material for: Podoconiosis: Clinical spectrum and microscopic presentations
Source: PLoS Negl Trop Dis. 2022 May 23;16(5):e0010057. doi: 10.1371/journal.pntd.0010057 (PMC9166354; doi:10.1371/journal.pntd.0010057)
Supplement: S2 Text — (DOCX) [file pntd.0010057.s003.docx]

**Annex C**

**Procedures and variables**

The skin biopsy was performed by the dermatologist (WE) or a surgeon in an area of the skin on the dorsum of the foot, which is the usual site of swelling of podoconiosis. The procedure was as follows: Irregular protrusions and skin lesions were not biopsied. After obtaining written informed consent, the skin was sterilised with an iodine swab anesthetized with a sub-epidermal injection of lidocaine (HCL 1% and Epinephrine 1:100,000). With a sterile 6mm skin punch, the skin sample was removed from the vicinity and with scissors from the underlying tissue, fixed in buffered formaldehyde and subjected to histopathologic processing. Patient was advised on home based wound care seen after 1^st^ week, 2^nd^ week and in one month. Follow up of patients for one month has showed no complication and no significant wound infection

Clinical data:

The following data were collected: socio-demographic characteristics (sex, age, marital status, education, level of completed education, occupation);associated risk factors(first degree family members diagnosed with podoconiosis, date of first seeking care for podoconiosis related complaints, foot washing in the last 24 hours, applied moisturizer in the last 24 hours, the age at which shoes were worn first, and whether shoes were worn consistently);clinical characteristics (duration of illness, staging, hyperkeratotic papules, presence of nodules, type of lymphoedema, presence of foot cracking, toe fusion, oozing, bad smell), podoconiosis-related complications (ulcers, number of episodes of ADLA, fever at the time of examination, burning (pain) at the time of examination, days of interruption of work during ADLA episodes, eczema on foot, fungal infection on foot).
