# Supplementary material for: Podoconiosis: Clinical spectrum and microscopic presentations
Source: PLoS Negl Trop Dis. 2022 May 23;16(5):e0010057. doi: 10.1371/journal.pntd.0010057 (PMC9166354; doi:10.1371/journal.pntd.0010057)
Supplement: Attachment — Submitted filename: Response to the reviewer WE.docx [file pntd.0010057.s004.docx]

Dear Reviewers

We are grateful for the instructive comments by the peer reviewer. We have accepted all their comments and used them to improve our manuscript. In addition, while revising our manuscript, we have corrected a few small spelling mistakes and typos. All changes in the revised manuscript are highlighted using the “Track Changes” feature in Microsoft Word. To respond for your comments and questions

1. Noting the assessment of participants consulting health care before the date of the their current presentation, might you consider including the estimate of how many consulted traditional healers for example?

A: As part of the questionnaire, participants were asked whether or not they had previously visited health facilities for treatment and at what age (and we summarised their replies in the manuscript). Unfortunately, we do not have information about which type of health provider the patients visited. No changes made.

2. Surgically puncturing the lymphoedematous limb which has potential to increase risk of ADLA and leaves behind hyperpigmentation, this hasnot been commented on. I assume this is not noted in your cohort? Might be worth mentioning.

A: Indeed, in this study, we observed no significant wound infections or other complications after one month of follow-up (mentioned in the annex). To accommodate the reviewer’s suggestion, we have added the following sentence in the methods section (study population and procedure): “In two previous studies on surgical wide excision (nodulectomy) for podoconiosis, we documented that wound healing was satisfactory, also in patients with lymphoedema. We therefore considered that the risk of complications such as wound infection, ADLA, or hyperpigmentation following 6mm skin punch biopsy would be low)

3. Also worth in the introduction stressing your previous study showing the safety and satisfactory healing of surgery in this patient group encouraging you that biopsy was appropriate and reasonable to do for research purposes in this setting.

A: We accepted this suggestion and added two sentences in the methods section (see reply to comment nr. 2)

4. healing and complication rate of your biopsies if can just to reassure/educate the lymphoedema community who otherwise would avoid trauma.

A: All patients received advice on skin care and wound healing was followed up in the 1^st^week, 2^nd^ week and one month after the biopsy procedure. No complications were recorded and the wounds healed without incidents (documented in the annex as part of the procedure).

5. In the abstract (line 37) the main conclusion expressed was the reduction in lymphatic vessels, however in results (lines 287) the main findings were dilated and ecstatic lymphatic vessels, also shown in table 4.

A: We observed both phenomena: the lymphatic vessels were dilated and ecstatic lymphatic and they were also reduced in number. To avoid confusion, we have rephrased the findings.

6. In my opinion, the description of the authors about the asymmetry of clinical manifestations of the disease may be confusing. Patients may have uni or bilateral affection; however, in the abstract it is said that 251 had podoconiosis in both legs and 104 patients had only one leg affected, while in lines 201-203 the authors describe 251 patients with both legs affected, and 38 with one leg podoconiosis

A: Thank you very much for pointing out the inconsistency. There was indeed a mistake in the abstract which we have now corrected: 251 patients had podoconiosis affecting both legs and 38 patients had podoconiosis affecting one leg. The title of table 2: I wonder if this might be better "clinical features distributed by stage" rather than "risk factors"?

A: Point well taken. We have reviwed the title of table 2

7. Information about Durbete town:

A: We have added the following sentence: “Durbete is a district in South Achefer zone, Amhara region, Ethiopia at an altitude ranging from 1914 to 2045 meters above sea level. The district is known as an endemic place for podoconiosis in the region.”

8. Lines 87-90: there is another paper that describes the histopathology of two patients with podoconiosis: Med Clin (Barc). 2015 Nov 20;145(10):446-51. doi: 10.1016/j.medcli.2014.12.020. PMID: 25726310.

A: Thank you for drawing our attention to this publication that we were unaware of. In the revised manuscript, we now refer to this paper in the introduction and the discussion.
